# Review of Soil Quality Improvement Using Biopolymers from Leather Waste

**DOI:** 10.3390/polym14091928

**Published:** 2022-05-09

**Authors:** Daniela Simina Stefan, Magdalena Bosomoiu, Annette Madelene Dancila, Mircea Stefan

**Affiliations:** 1 Department of Analytical Chemistry and Environmental Engineering, Faculty of Chemical Engineering and Biotechnologies, University Politehnica of Bucharest, 1-7 Polizu Street, 011061 Bucharest, Romania; daniela.stefan@upb.ro (D.S.S.); madelene.dancila@upb.ro (A.M.D.); 2 Pharmacy Faculty, University Titu Maiorescu, 22 Dâmbovnicului Street, 040441 Bucharest, Romania; stefan_apcpm@yahoo.com

**Keywords:** biopolymers, leather waste, soil, fertilizers, industrial crops

## Abstract

This paper reviews the advantages and disadvantages of the use of fertilizers obtained from leather waste, to ameliorate the agricultural soil quality. The use of leather waste (hides and skins) as raw materials to obtain biopolymer-based fertilizers is an excellent example of a circular economy. This allows the recovery of a large quantity of the tanning agent in the case of tanned wastes, as well as the valorization of significant quantities of waste that would be otherwise disposed of by landfilling. The composition of organic biopolymers obtained from leather waste is a rich source of macronutrients (nitrogen, calcium, magnesium, sodium, potassium), and micronutrients (boron, chloride, copper, iron, manganese, molybdenum, nickel and zinc), necessary to improve the composition of agricultural soils, and to remediate the degraded soils. This enhances plant growth ensuring better crops. The nutrient release tests have demonstrated that, by using the biofertilizers with collagen or with collagen cross-linked with synthetic polymers, the nutrient release can be controlled and slowed. In this case, the loss of nutrients by leaching into the inferior layers of the soil and ground water is minimized, avoiding groundwater contamination, especially with nitrate.

## 1. Introduction

The leather industry is continuously increasing, due to the increased demand for finished leather products and meat for human consumption. This generates large quantities of waste (fleshings, hairs, shavings, dust, liquid waste which contains the tanning agent) from different steps of leather processing. These wastes have a rich content of proteins (collagen, gelatine and keratin) [1,2]. Recent strategies demand a transition towards zero landfill and waste in leather production by reusing the leather waste as secondary raw material [3].

The most fertile layer of the soil is the topsoil, which is the top layer of the soil and is rich in microorganisms, minerals and humus. This layer is considered the best location for crop development.

A soil is considered to be contaminated when a moderate increase in substances occurs, substances that are not harmful for a plant to grow at this stage. Soil degradation consists of the action (simultaneous or not) of several physical, chemical and biological factors on the soil. Intensive agriculture, climate change, acid rains, water shortage caused by drought, and accelerated growth of the population all consist of elements that determine the acceleration of soil erosion and nutrient depletion. The soil is continuously exposed to physical erosion by wind and water, a fact that causes the loss of fertile topsoil. The chemical factors consist of acid rains, accidental chemical pollution, and excessive use of chemical fertilizers and pesticides. Moreover, poor farming activities can lead to a decrease in microbial activity in the soil. Therefore, it is obvious that there is a necessity for soil quality improvement to obtain at least the original state of fertility, and productivity of agricultural soil.

Long-term prediction of soil fertility and biodiversity can be approximated using a series of indicators such as: soil structure, soil pH, soil erosion, soil organic matter, phosphorous and potassium content, humidity, etc. These parameters give both qualitative and quantitative information about the possible behavior of soil, over a more extended period [4]. Among these factors, soil organic matter and humidity play a key role in keeping the nutrients available and preventing soil erosion. In the countries with warm temperatures and dry winters, the loss of organic matter is accelerated by the enhanced decomposition of crop residues [5]. The pH controls the chemical and biochemical processes, by enhancing or not enhancing the availability of some nutrients to the plants, and by increasing micro-organism activity. The plant itself can change the soil pH value, e.g., vegetables such as soybeans lower the soil pH [4]. Soil structure is characterized by the porosity and pore size distribution in the soil layers. Soil compaction reduces crop productivity because the plant roots develop and grow in the pores of the soil [6]. An extensive study of Luvisoil type agricultural soil modification, when applying biochar, oyster shells, biopolymers (synthesized from lignin or starch), or synthetic polymer (polyacrylamide) was made by Awad et al. [7]. The authors have shown that the application of the polyacrylamide solely increased the percentage of macroaggregates (1 to 2 mm). The addition of oyster shell to biochar or biopolymer increased the percentage of microaggregates (size below 0.25 mm), having a positive effect on soil quality.

**Figure 1:**
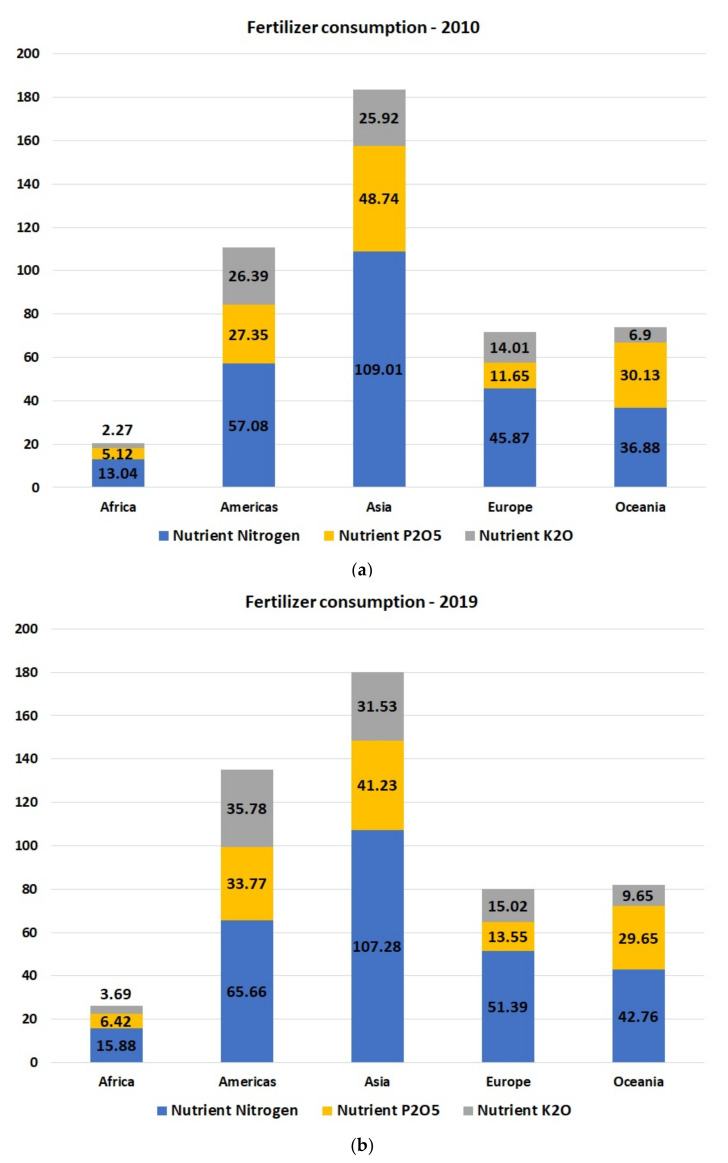
Fertilizer consumption expressed as nitrogen, phosphate, P_2_O_5_, potassium K_2_O, in the last 10 years (**a**) 2010, (**b**) 2019 [12].

For a continuously increasing population estimated to achieve 11 billion people by the end of this century, it is mandatory to conserve the soil fertility, and to ensure food quality and people’s safety [4,8,9,10]. The most common strategy to increase crop production is fertilization [11]. When looking at the statistics about fertilizers consumption worldwide, in the last 10 years, a trend of continuous increase in fertilizer consumption can be observed (Figure 1) [12]. The demand for healthier agricultural products is continuously increasing as a result of customer awareness increase. Kilic et al. compared the results obtained from GAP farms (Good Agricultural Practices program) with those obtained from farms not included in this program regarding the use of chemical fertilizers, pesticides, the yields and the gross profit [13]. The scope of this program is to reduce the use of pesticides and harmful substances with negative environmental impact which cause health problems, in view of making agriculture more sustainable.

Polymers are natural or synthesized large molecules made by linking repeating units, monomers; they are characterized by different physical and chemical properties than their constituent monomers. Biopolymers are polymers produced by living organisms (such as plants and animals), and they are a renewable resource of polymers. The repeating units can be nucleic acids, saccharides and amino acids having either linearly or branched structure molecules, and the formed biopolymers can be polysaccharides (carbohydrates, starch, cellulose), proteins (collagen, keratin, gelatine), polynucleotides (DNA, RNA) [1,2]. Natural polymers are used for medical and pharmaceutical applications [14,15,16,17,18,19,20], food additives [21], and, in recent years, they received attention for agricultural applications because of their particular properties such as: biodegradability, biocompatibility, non-toxic, bioactivity and hydrophilic character [22]. Both natural [23,24,25,26,27,28] and synthetic polymers [29,30,31] have reportedly been used to stabilize soils.

Over time, a number of studies have been dedicated to synthesizing and improving the characteristics of the fertilizers that are used to maintain the balance between human needs for consumption and natural available resources. One of these substances is superabsorbent hydrogels (SAHs), which have been proved to be beneficial for plant growth and soil health, and, consequently, have extensive applications in the agricultural field. The SAHs are polymeric materials that have the capacity to retain a large amount of water and nutrients and to slowly release the water along with the nutrients, to respond to the plant demand. The SAHs can be natural (collagen, gelatine), synthetic, or combined polymers (cross-linked). The natural polymers are easily biodegradable but have low functional properties needed for agricultural properties. Therefore, it is preferable to use natural polymers cross-linked with synthetic polymers, to combine the properties of the two categories of polymers [8,9,32]. Soil crusting impedes seedlings and accentuates water runoff. Polyelectrolytes (e.g., based on polyacrylamide), which are synthetic polymers, improve the soil quality by preventing the crust formation in a critical period between plant seeding and emergence, increasing the resistance to water and air erosion of soils, and improving the soil permeability by enhancing the formation of hydro-stable structural aggregates [8].

It has been reported that collagen SAHs obtained from leather waste have a swelling capacity of more than 2000% by weight [33,34], while gelatine SAHs synthesized from chicken waste have a swelling capacity of more than 800% by weight [35]. Another SAH category is the chitosan-based one. Unlike collagen and gelatine that are recovered from leather waste, chitosan can be extracted from squid bones, crustacean shells, and insects. Essawy et al. studied the cross-linking of chitosan with cellulose, to improve the resistance to acidic soil conditions, and increase water retention capacity [36].

The most used chemical fertilizer as a nitrogen and carbon source is urea, which has a relatively low cost [37]. However, only a small percentage of the applied urea will be effectively used for crop growth, because of urea’s high volatility and solubility in water [38]. Urea hydrolysis produces ammonia, which, in turn, has a negative impact on seed germination, respectively, on seedling growth in soil. [39]. Another disadvantage is ammonia volatilization and nitrite accumulation in soils, which can be further leached and cause environmental problems. To overcome these disadvantages, new methods which consist of the controlled release of the fertilizer were developed. These methods consist of the deposition of organic/inorganic functional materials, coating with polymers, encapsulation in matrices, and copolymerization via immolable bonds [40,41,42,43,44].

Many industries, including the leather industry, produce large quantities of wastes that are rich in organic matter [11,45,46,47]. The skin consists of three main layers: the epidermis, dermis and hypodermis; the dermal layer, representing 85% of total skin thickness, is the main layer and consists of type I fibrillar collagen [48]. Leather waste is an important source of raw materials such as protein and gelatine, providing elements such as nitrogen and carbon essential for plant development. Biopolymer-based fertilizers can be applied either for the purpose of degraded soil rehabilitation or for crop quality and quantity improvement [1,49,50].

Obtaining biodegradable polymers with application in agriculture is multidisciplinary research that involves the steps of recovering the collagen or the gelatine from leather waste materials (obtaining the so-called protein hydrolysate, PH), enrichment of collagen/gelatine with P and K nutrients, functionalization with synthetic polymers and testing the obtained bio-fertilizer on different types of crops/soil type/application rate [51,52]. The implementation of this method also requires an economic analysis besides scientific acceptance.

Leather processing involves multiple preparatory steps: curing, soaking, painting, liming, fleshing, de-liming, degreasing, tanning, splitting, shaving, finishing, etc. [53,54] During these steps, about 35–40% of the raw material is found as waste in various stages of processing [55,56].

Depending on its provenance, leather waste can be classified into two main groups:

(a) untanned leather waste from the processing of raw and gray leather: wax (hypodermic layer) and gelatine skin (fringes and cuttings from the shaping of the leather contour), which represents the dermal layer without epidermis, hypodermis and hair;
(b) tanned leather waste from the processing of tanned and finished hides, from the leather footwear and clothing industry (tanned leather and finished leather).

Several proteins of high value can be recovered from leather waste: gelatine and collagen are the so-called protein hydrolysate part of the waste and the tanning agent (mostly chromium) that can be reused [51,52,57]. This is in line with the circular economy strategy allowing the recovery of valuable products that otherwise would be not only a loss of raw materials but also harmful to the environment. Moreover, the extraction of keratin from hairs with the purpose of using the keratin hydrolysate in agriculture has also been reported [58].

The protein hydrolysate is a mixture of peptides and amino acids that can be obtained either by chemical (basic or acid) or by enzymatic hydrolysis of leather waste [59,60].

**Figure 2:**
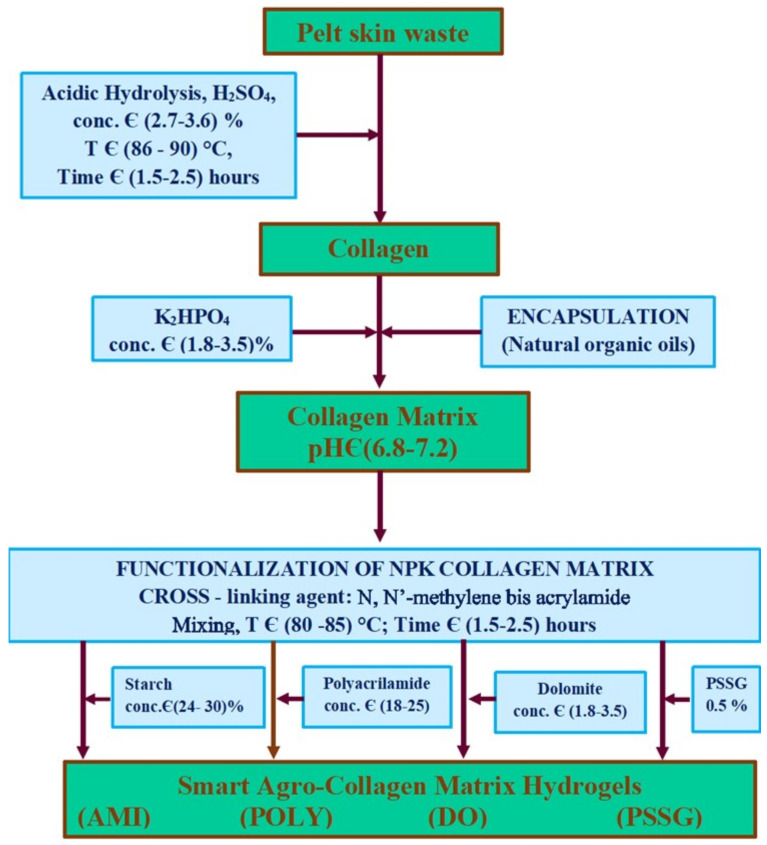
Technology scheme for obtaining smart-fertilizers by using acid hydrolysis, readapted from [77]; CH—collagen hydrolysate, Ref—CH—collagen hydrolysate with nutrients encapsulated as reference sample, PSSG—Ref—CH functionalized with P(SSNa—co—GMAx) copolymer, POLY—Ref—CH functionalized with poly-acrylamide, AMI—Ref-CH functionalized with starch, AMI—Ref-CH functionalized with dolomite).

Traditionally, chemical hydrolysis is achieved with strong acids (e.g., sulphuric acid, phosphoric acid) or alkaline bases and around 80 °C, allowing chromium removal without destroying the collagen tissue [61,62]. These methods are especially employed when it is necessary to remove the tanning agent (chromium). During the chemical hydrolysis part, the amino acids and peptides are lost; to overcome this disadvantage and for the non-tanned leather waste, enzymatic hydrolysis can be applied [63]. The enzymatic hydrolysis uses specific enzymes and lower temperatures (<60 °C) [64,65]. Due to the health and environmental problems generated by chromium use in the tanning step, low chromium methods [66] and eco-friendly alternatives have been developed consisting of using vegetable tannins [67,68], aluminum salts [69,70], titanium salts [71], combined vegetable-aluminum tanning agents [72], 4-(4,6-dimethoxy-1,3,5-triazin-2-yl)-4-methylmorpholinium chloride [73] or amino-acids [74,75,76]. Hide and leather waste characteristics and processing have been reviewed in a previous publication [60]. The methods of collagen recovery and chromium removal from tanned waste have been extensively discussed; therefore, this review is the second part and presents aspects related to the use of the extracted collagen as fertilizer. In Figure 2, the technology of obtaining a collagen-based fertilizer by using acid hydrolysis is exemplified [77]. For more details on this topic, the readers are advised to first lecture the article presenting the methods for leather-based fertilizers synthesis [60].

## 2. Comparison among Biopolymer-Based Fertilizers Obtained from Leather Waste and Other Types of Fertilizers (Chemical Fertilizer, Compost, etc.) Applied for Crop Growth

Biopolymer-based fertilizers have the advantage of retaining large quantities of aqueous solutions, along with the slow release capacity of the water and of the nutrients, over extended periods of time [33,34,35]. On the contrary, chemical fertilizers do not have the capacity to retain water and therefore ensure a constant humidity level in conditions of drought. Moreover, chemical fertilizers are known to release their nutrients in the first few days after the fertilization, ensuring fast development of the plant in this period [78].

Collagen and gelatine are important resources of nitrogen and carbon. The main amino acids found in the composition of collagen powder, extracted from chromium leather scrap waste, are: aspartic acid, threonine, serine, glutamic acid, glycine, valine, isoleucine, tyrosine, phenylalanine, histidine, arginine, and proline [79].

Commercial hydrogels have been tested as a source of nutrients and moisture conservation, in the Semi-Arid Zone of Kongelai (Kenya), for the cultivation of *Cajanus cajan* [80]. The experiments were conducted both in a nursery and in a field, and the results showed that the use of hydrogels retards plant growth in nursery soils, but improves growth in the field by increasing the soil moisture.

**Figure 3:**
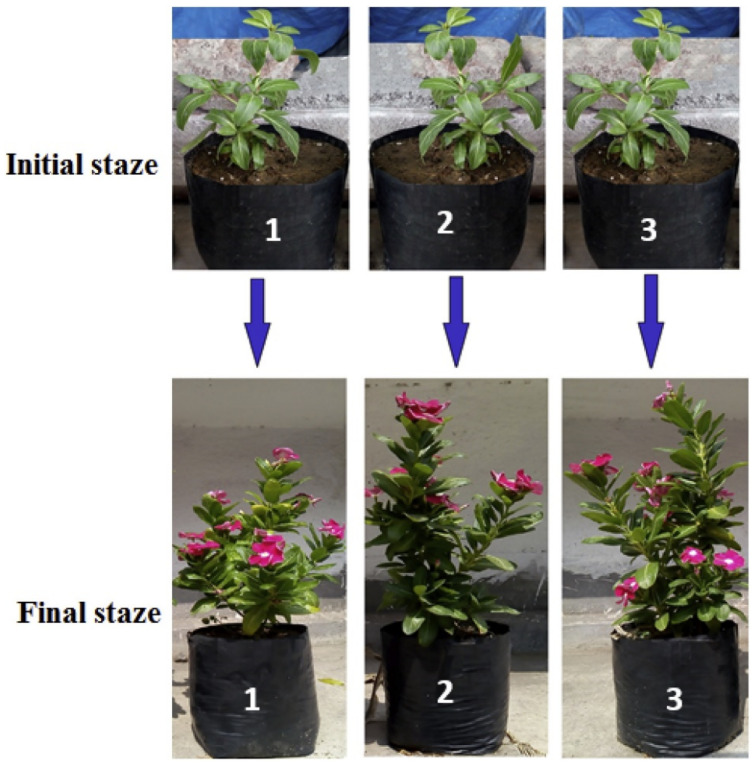
Plant growth comparison: (**1**) Control plant without fertilizer; (**2**) plant with collagen NPK fertilizer; (**3**) plant with commercial fertilizer; final stage is the 80th day after the soil fertilization. Reprinted with permission from ref. [52]. 2022, Elsevier.

Majee et al. synthesized a biopolymer-based fertilizer, recovering the collagen from tanned leather waste, and enriching this material with poultry bone meal as a source of phosphorous, and with water hyacinth ash as a potassium source [52]. This fertilizer was applied to the *Catharantus roseus* (Madagascar Periwinkle) plant, and a comparison was made with a commercial fertilizer, or with a plant without fertilizer. The results for the three cases are shown in Figure 3 together with the initial stage where the plants had no fertilizer applied. The authors compared the plant growth by considering plant length and diameter, leaf size, flower size, and total number of flowers. Both chemical and biopolymer-based fertilizers provide nutrients for good plant development, although in the initial stage, the growth of the plant with chemical fertilizer was accelerated, due to the fast nutrient release.

These results are in agreement with a later study conducted by Majee et al. when a combined polymer-potato peel biochar fertilizer was synthesized and tested [81]. The source of phosphorous and potassium was the potato peel biochar, obtained at lower temperatures, to conserve several functional groups useful in agricultural applications.

The test experiments were conducted on *Abelmoschus esculentus* (okra plant), cultivated in pots containing soil without fertilizer, with biopolymer-based fertilizer, or with chemical fertilizer.

A comparison between green manure, mineral N-fertilizer, and biopolymer-based fertilizers obtained from leather waste, was made for the tomato crop [82]. Two types of biofertilizers were used: 5% N and 8% N, respectively. The results showed that the 5% N biofertilizer does not provide sufficient N for the plant to grow, while the 8% N gave similar results to the mineral fertilizer.

Table 1 presents different types of biofertilizers synthesized from leather waste that were tested on various plants, together with the applied amendment rates and the type of soil.

Farneselli et al. (Table 1), conducted an extensive study, investigating for 2 years the efficiency of fertigation treatments on tomato crops using poultry manure, by-products from the leather factory, organic by-products from the leather factory, and mineral liquid fertilizer (7.5% NO_3_-N + 7.5% NH_4_-N + 15% urea, radicon N30).

Fertigation is applied at rates of 100 and 200 kg N/ha, in 10 splits (2 times/week for 5 weeks), according to the expected nitrogen uptake rate for tomato processing throughout the growing season. It was found that fertigation treatments, using mineral and organic fertilizer by-products from the leather factory in doses of 200 kg N/ha almost always caused luxury N consumption, for both situations, in the first year and a deficit in the second year for organic by-products from the leather factory. Reducing N rates, both for the mineral and for the organic one to 100 kg N/ha, ensured optimal N status for the main part of the crop cycle with a slight deficiency of growing at the end of the second year. The effect of mineral and organic by-products from the leather factory is similar.

The use of poultry manure and by-products from the leather factory is similar to other fertigations that used doses of 100 kg N/ha. In the first year of fertilization, the nitrogen uptake was 27% higher than the second year. N uptake with fertilization with mineral fertilizer at a dose of 200 kg N/ha was significantly higher than any other treatments but much larger than the optimum N status [82].

This study shows that, although mineral fertilizers are particularly effective, a well-known fact, they can be replaced with fertilizers derived from leather waste.

Table 2 gives the nutrient composition of several mineral fertilizers and leather-based biofertilizers.

It can be seen that the classic fertilizers urea, ammonium nitrate and NPK have a very high nitrogen content (over 21%) and can reach up to 46%. NPK fertilizers also contain macroelements such as phosphorus and potassium, which gives them extra efficiency in terms of plant growth and development. Conventional fertilizers release nutrients quickly, and consequently, losses are significant. Ammonium nitrate loses nitrogen the fastest, followed by urea, ammonium sulfate and Floranid (IBDU) [40].

Fertilizers obtained from leather factory by-products contain between 5 and 8% of N, while those obtained from hydrolysates of leather waste have a nitrogen content of up to 16%. These fertilizers have a complex composition containing, in addition to nitrogen, other macronutrients, such as phosphorus and potassium, that can be introduced by mixing, but also micronutrients absolutely necessary in plant growth and development. They also have a high carbon content in biodegradable organic compounds, that can grow soil fertility [82,83,84,85].

Due to the complexity of the composition, the fertilizers derived from leather waste have an action with a much wider spectrum, that aims at a positive action both on the growth of the plants, and on the improvement of the soil quality. Conventional mineral fertilizers are effective, but their action is one-sided and can be characterized by point-to-point hits.

Several alternatives to the use of the leather waste hydrolysate as fertilizers are the composted or vermicomposted leather waste. These methods have the advantage of lowering the carbon to nitrogen ratio, providing more nitrogen necessary for plant growth [88,89].

Silva et al. studied the use of tannery sludge for the cultivation of ornamental *Capsicum* plants [46]. The compost was prepared from tannery sludge, mixed with agricultural waste (sugarcane straw, and cattle manure, “carnauba” straw, and cattle manure, respectively) in different ratios. Results have shown that, when replacing the inorganic fertilizer with these composts, there was a significantly increased number of leaves and fruits, as well as a higher content of chlorophyll in the leaves. However, the concentrations of Cu, Cd, Cr, Zn, Pb, Ni, Mo, and Mn increased in soil, because of soil amendment with composted tannery sludge. This suggests that tannery waste is a good option when it is necessary for the amendment of soils to grow ornamental plants and not recommended for plants and crops intended for human consumption. A more recent study of leather biodegradability showed that the quality of the compost is influenced by the nature of the tanning agents (chromium or titanium salts) and that biodegradation is a complex process that could be achieved in the presence of food wastes [71]. Altogether, the titanium tanned hides were more biodegradable than the chrome tanned hides and vegetal tanned hides.

Vermicomposting supposes the use of several earthworm species such as: *Eisenia fetida*, *Eisenia andrea*, *Eudrilus eugeniae,* for the conversion of different waste types (including leather waste), into a product useful for soil amendment [90,91,92,93,94,95,96,97]. Ravindran et al. (2019) studied the amendment of soil with vermicompost hydrolyzed tannery animal fleshing on the growth and yield of commercial crop tomato plant (*Lycopersicon esculentum*) [88]. The quality of tomato fruit was assessed from the point of view of its size, weight, tomato juice pH, ascorbic acid, total sugar content, etc. These parameters indicated that plant growth, yield, fruit quantity, and nutrients in fruits were higher when the soil was treated with the leather waste vermicompost, compared to the control sample. Tannery waste vermicomposting, like composting, does not involve chromium recovery prior to its use in agriculture. Over time, this causes chromium to accumulate in the soil and is a major drawback. One method to reduce the chromium impact on the crop, and also on the earthworms (as chromium is toxic to earthworms), consists of the mixing of tannery sludge with other materials (e.g., manure) to reduce the concentration of chromium ions [89].

Vermicompost tannery sludge was compared with the conventional NPK fertilizer, and control sample (soil without fertilizer), in the cultivation of sweet pepper [98]. The addition of vermicompost stimulated the plant growth and enhanced the production of more fruits per plant (one fruit harvested per plant, for NPK fertilizer, vs. up to three fruits harvested per plant for vermicompost). The authors found similar chromium contents in all the fruits (control sample, NPK conventional, and tannery sludge vermicompost), indicating that there is not a major contamination of fruits with chromium, but did not study a possible chromium accumulation in time, in the soil amended with vermicompost tannery sludge.

A ten-year study on the early application of composted tannery sludge showed that soil properties change and that elements accumulate: organic matter, N and K content, increased over the 10 years of the study, showing a positive effect of this treatment [99]. However, the soil pH and chromium content also increased, which is not beneficial for soil used for agricultural purposes. During the experiment, it was found that chromium content increase took place mainly in the first 5 years and remained almost constant in the next 5 years. Another disadvantage of composted tannery sludge application was that the enzyme activity decreased. This affects a lot the complex process of transforming organic compounds into assimilable subunits (sugars, amino acids, NH^4 +^, PO_4_ ^−3^).

A biofertilizer obtained from titanium tanned leather waste, by combined chemical-enzymatic hydrolysis (the so-called “wet white leather”), was tested for pea crop growth [82]. After the chemical hydrolysis, two phases are obtained: the liquid phase containing the collagen recovered, and an unhydrolyzed solid phase called “titanium-containing sludge”. The two phases are separated. The resulted sludge is subjected to enzymatic hydrolysis, in the presence of lipase, cellulase, amylase and protease. The titanium salt was recovered for reuse, as a tanning agent. The protein hydrolysate is modified by chemical cross-linking with other polymers such as: polyacrylamide, acrylic polymer, maleic polymer, cellulose or starch. The cross-linking process gives the fertilizer resistance to water dissolution. Moreover, the addition of polyelectrolyte-type polymers improved the soil properties, by increasing the resistance to water and wind erosion for the soil located on slopes. It also prevents crust formation after sowing, which is essential for plants with small seeds [8,100]. The study of the amended soil indicated that the fertilizer was efficient not only for the peas’ growth, but also for the remediation of the soil quality.

**Figure 4:**
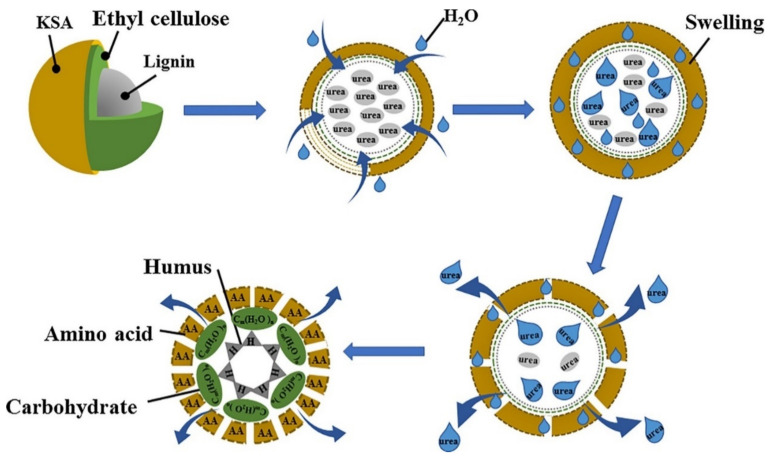
Schematic representation of keratin-based fertilizer nutrient release. Reprinted with permission from ref. [78]. 2022, Elsevier.

Keratin-based fertilizer has proved its efficiency in remediation of the soil contaminated by heavy metals, by fixing the chromium(III) contained in the soil [77]. The keratin was extracted from the waste bovine hair, and cross-linked with acrylic acid and N,N-methylene bis acrylamide, to form a keratin-based superabsorbent material. The core of the fertilizer spheres, made by a mixture of lignin powder and urea particles, is covered by an ethyl-cellulose layer, and finally, by a superabsorbent material (Figure 4). This fertilizer was tested in the wheat growth and gave better results than urea.

**Figure 5:**
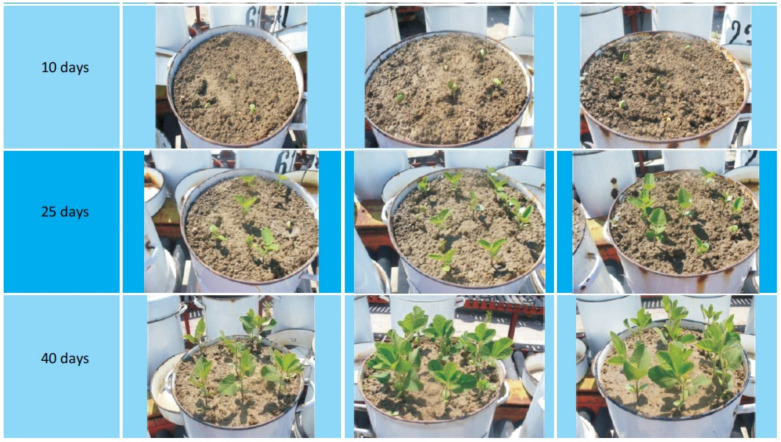
Soybean growth comparison (left—no fertilizer; middle—10 kg collagen NPK fertilizer/m^2^; right—20 kg collagen NPKfertilizer/m^2^) [83].

Constantinescu et al. tested a fertilizer obtained using the collagen recovered by alkaline hydrolysis of untanned leather waste using K_2_HPO_4_·3H_2_O [83]. The fertilizer was applied at two rates: 10, 20 kg/m^2^, respectively. The soil was amended with fertilizer before planting the seeds, to stimulate the processes of germination, seedling growth, deep rooting, and rigorous plant development. The growth of the plants was compared after 10, 25, and 40 days (Figure 5). It can be seen that there is almost no difference between the two rates of fertilizer (middle and right pots).

**Figure 6:**
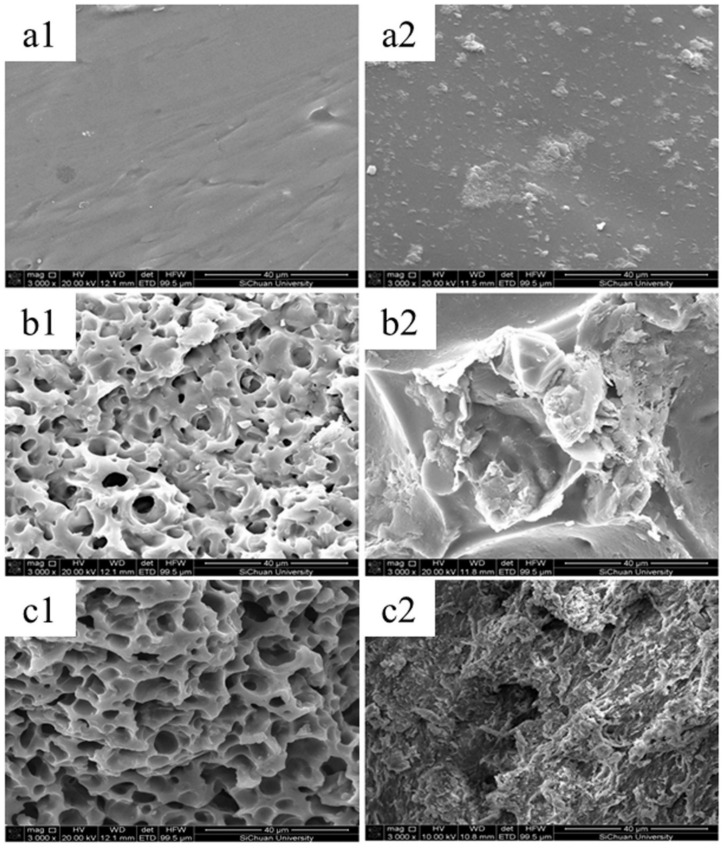
SEM images of control (**a**) and collagen-based fertilizer (**b,c**) samples before (**1**) and after (**2**) degradation in soils (**a,b**) and in *Ensifer* sp. Y1 medium (**c**). Reprinted with permission from ref. [33]. 2022, Elsevier.

Hu et al., investigated how the structure of leather-based biofertilizer changes in soil after 60 days, by using the scanning electron microscopy technique [33]. The fertilizer porosity is an important parameter, as higher pore diameter values determine higher surface area per volume ratio, a fact which enhances the swelling rate and biodegradability [34]. The biodegradability tests were performed in the presence of *Ensifer* sp. Y1 bacterium, isolated from the soil. The selected samples were: control hydrogel in soil, fertilizer in soil, and fertilizer in *Ensifer* sp. Y1 medium (Figure 6). It can be seen that the fertilizer samples initially have a microporous structure that is lost after 60 days in soil, while the control hydrogel is slightly degraded. The presence of large colonies of *Ensifer* sp. Y1 enhanced the fertilizer biodegradation.

Nogueira et al. used tanned leather waste (wet blue leather) to synthesize a N_collagen_PK biofertilizer tested for the growth of rice plants [101,102]. The chromium tanning agent was extracted according to the method presented in a previous study, which proved to be very efficient, recovering up to 99.6% of chromium contained in the waste [103,104]. The fertilizer was applied on a typical dystrophic Yellow-Red Latosol, clayey texture, Oxisol and its fertilization was compared with a commercial NPK fertilizer and urea, enriched with P and K, respectively. The biofertilizer showed activity similar to the urea enriched compound in the growth of rice plants, and slightly lower than the commercial NPK fertilizer.

## 3. Nutrient Releasing Processes

Fertile soils contain different inorganic mineral particles (sand, clay, silt); assimilable and non-assimilable organic matter; living organisms (earthworms, insects, bacteria, fungi), water, gases (O_2_, CO_2_, N_2_, NO_x_, CH_4_). The inorganic materials are involved in the retaining processes of cations through ion exchange, and of anions and organic compounds through sorption (surface) reactions [105]. The temperature, soil pH, and humidity control the molecules/ions interchanges between soil phases (solid, liquid and gaseous) and soil biological activity. The living organisms contribute to topsoil regeneration by humus formation. They decompose the organic matter into assimilable forms, increase the soil porosity (and consequently, the aeration), and help the movement of organic matter and residues within the topsoil [106,107,108]. The microorganisms transform the nitrogen present in the fertilizers into nitrate, through the nitrification process. It has been reported that soil microorganisms are able to increase the carbon sequestration capacity, as CO_2_ concentrations are continuously increasing [109]. Adding organic fertilizers helps beneficial microorganism development (*Fimicutes, Chloroflexi, Bacillus* and *Actinomadura*), increases enzyme activity (like sucrase enzyme), and increases *Fusarium* and *Phytophthora* pathogen mortality [107]. Organic matter contained in the biofertilizers is a resource of C, N, P, and S nutrients. The K and supplementary P are provided by enriching the biofertilizer with inorganic K and P.

Although there is great interest regarding the controlled release fertilizers [44,110,111,112], few studies have been dedicated to studying the nutrient release in leather-based biopolymers [77]. Most studies that have been published present the synthesis of the biopolymer fertilizer and its testing for different crops, being lesser dedicated to the nutrient release mechanisms. For a keratin-coated urea fertilizer, the schematic representation of nutrients’ release steps is given in Figure 4 [78]. Once spread in the soil, the fertilizer particle starts to retain water from the soil until reaching the swelling equilibrium. The water passes through the ethyl cellulose and lignin layers, starting to dissolute the urea. The two layers act as a barrier to water passage, and they are meant to delay the penetration of water [113,114]. Dissolved urea diffuses to the exterior of fertilizer particles through the perforated layers of ethyl cellulose and lignin and is slowly released into the soil. The superabsorbent material and the ethyl cellulose and lignin layers are further degraded, to provide more nutrients (amino acids, carbohydrates and humus), under the action of soil microorganisms. The nutrient release experiments performed with uncoated urea and with keratin-based fertilizer showed that about 83% of uncoated urea was released in the first 24 h [78]. On the contrary, the biopolymer fertilizer released about 70% of encapsulated urea over 28 days, demonstrating its excellent performance in controlling the nutrient release.

**Figure 7:**
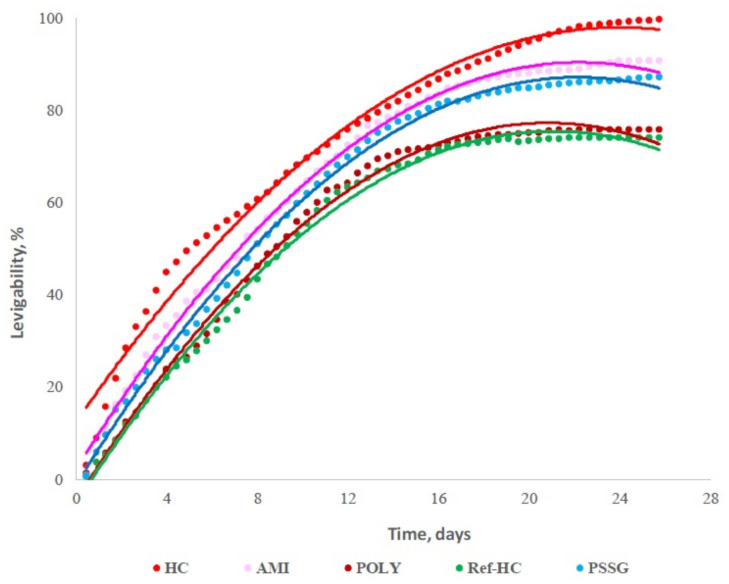
Oxidable nutrient release (organic and inorganic), in water for different collagen-leather-based biopolymers [77]; HC—collagen hydrolysate, Ref—HC—collagen hydrolysate with nutrients encapsulated as reference sample, PSSG—Ref—HC functionalized with P(SSNa—co—GMAx) copolymer, POLY—Ref—HC functionalized with polyacrylamide, AMI—Ref—HC functionalized with starch.

The release of oxidable compounds (organic and inorganic) in water for different types of collagen-leather-based biopolymers has been presented by Stefan et al. [77]. Oxidable compounds released from tested fertilizers over a period of one month is shown in Figure 7. The release degree was evaluated in dynamic conditions, by the determination of chemical oxygen demand (CODMn) [115].

Collagen hydrolysate (HC) is practically completely degraded during chemical oxidation (>99%). Therefore, collagen cross-linking with a more stable polymer in aqueous conditions is needed. Among the functionalized biopolymers, collagen cross-linked with starch (AMI) has the highest nutrient release degree, over 25 days—namely about 90%. The lowest nutrient release is given by collagen functionalized with polyacrylamide (POLY). This suggests that synthetic polymers give a more stable structure to the fertilizer, slowing down the nutrients’ release for a longer period.

Experiments of the nutrients’ release in soil amended with NPK fertilizer without superabsorbent polymer showed a total release of nutrients (N, P, K) in the first 4 days [36].

**Figure 8:**
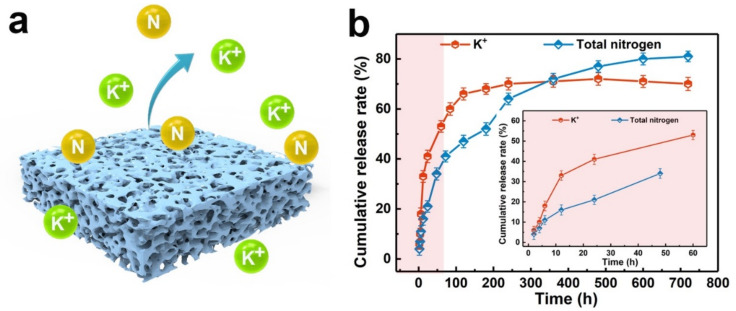
Biofertilizer synthesized from collagen-leather waste cross-linked with acrylic acid (AA) and maleic anhydride (MA), 15% leather waste hydrolysate: (**a**) porous structure; (**b**) N and K release in water. Reprinted with permission from ref. [33]. 2022, Elsevier.

Hu et al. tested the nutrients’ release from a porous collagen-leather-based biofertilizer, over a more extended time period of 34 days [33]. About 45% of the total nitrogen and 30% of K is released in the first 2 days; the rate of K release is more accelerated, compared with N release—which is gradually released in time. This can be explained by the fact that the N from collagen is made accessible to the plants by biodegradation in the presence of microorganisms, and this is a slower process, compared to K dissolution in water (Figure 8).

## 4. Use of Biopolymers for Soil Remediation and Stabilization

Over recent years, there was an increasing interest in recovering biopolymers from different types of materials and wastes, to test them for soil remediation purposes. Among these biopolymers there are: agar gum, which is a polysaccharide extracted from of *Rhodophyta* (red algae), such as *Gelidium, Gracilaria*, and *Pterocladia* [116,117]; guar gum, which is a neutral polysaccharide extracted from the seeds of the leguminous shrub *Cyamopsis tetragonoloba* [118,119,120]; gellan gum, which is an anionic polysaccharide made by microbial fermentation of *Sphingomonas elodea* [121,122]; dextran, which is a group of glucose polymers made by lactic acid bacteria such as *Leuconostoc mesenteroides* and *Streptococcus mutans* from sucrose [123,124,125,126], xanthan, which is a polysaccharide biopolymer produced by *Xanthomonas campestris* [127,128,129,130,131,132,133,134]; chitosan, which is a polysaccharide extracted by alkaline hydrolysis of crustacean shells, insects, squid bones [135], starch -which is composed of monosaccharides found in seeds, grains, and roots of plants (maize, rice, wheat, corn, potatoes, cassava, etc.) [116,136,137]; casein, which is a phosphorous protein biopolymer contained in milk products, and is used for soil remediation, due to its hydrophobicity [138,139,140].

Dang et al. studied the use of graft copolymer extracted from leather solid waste for its application in chemical sand-fixation [141]. The by-product gelatine was extracted from the leather solid waste by alkaline hydrolysis, and, in a second step, the graft copolymer was synthesized by free-radical copolymerization with acrylamide and acrylic acid. The final product was tested and proved to have good water retention capacity, good biodegradability, and sand stabilization properties, due to the formation of adhesion forces among the copolymer and the sand particles.

Soil degradation (physical, chemical and biological) represents the loss of its productivity, following the action of natural and anthropogenic factors. The good quality of agricultural soils can be severely affected by phenomena such as drought, erosion, salinization, acidification, alkalinity or compaction [83]. Degradation of agricultural land takes place also through contamination/pollution processes with heavy metals such as: iron, manganese, copper, zinc, lead, cadmium, chromium, cobalt, nickel [46,142]. Soil restoration consists of the application of remedial methods, to obtain higher soil fertility and productivity, or at least a state closer to the initial one. These methods aim to improve the soil structure, microorganisms density, nutrient density, and overall carbon levels of soil. Therefore, soil quality can be improved by maintaining the humus layer, increasing microorganisms populations and biological diversity, as well as by reducing the use of chemicals (fertilizers, pesticides, herbicides). Soil degradation leads to a vicious cycle: low crop yields determine malnutrition, social disorders and unequitable distribution of wealth; this gives low agricultural resources needed to remediate the soil quality, thus inducing more severe soil degradation. Moreover, the soil quality is closely related to the environment quality, since degraded soil is an indicator of other environmental issues (such as water contamination, poor biodiversity, and dust in the air).

**Figure 9:**
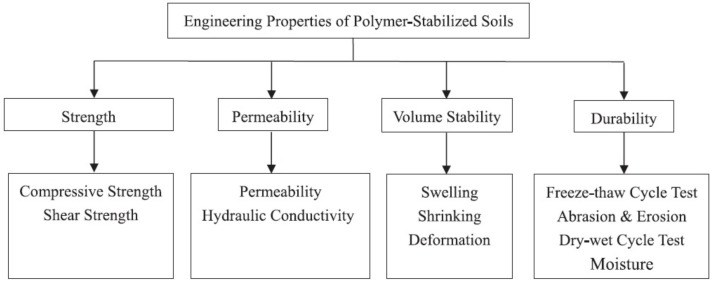
Methods to evaluate the effectiveness of soil stabilization using polymers. Reprinted with permission from ref. [31]. 2022, Elsevier.

The aim of soil stabilization and remediation is to improve its mechanical properties, provide a proper quantity of nutrients, and regenerate microorganism populations. Huang et al. reviewed the use of biopolymers, geopolymers (inorganic polymers with different Si-Al backbone structures), and synthetic organic polymers for soil stabilization [31]. The main properties that are taken into account to evaluate the quality of polymer-stabilized soil are presented in Figure 9.

Among the synthetic polymers employed for soil stabilization is polyacrylamide, which is also used in the copolymerization of collagen to produce biofertilizers. The PAM was applied to a wide range of soil types: silty gravel, clayey sand, clayey gravel [143,144,145,146].

Tingle et al. tested soils treated with different types of polymers and found a significant drop in strength under wet conditions [147,148]. The polymer addition improved the strength, compared to untreated soil.

Polyacrylamide alone applied to agricultural Luvisol soil type increased the proportion of large macroaggregates (1–2 mm), while the amendment with biochar, biopolymer and oyster shells increased the portion of microaggregates (<0.25 mm). The addition of biochar, biopolymer and oyster shells was found to contribute to the increase in the biological activity, by increasing the levels of leucine aminopeptidase and chitinase [7]. This was confirmed by higher NO_3_^−^ concentrations, leucine aminopeptidase being involved in the N-cycle [149], whereas chitinase is a measure of increased fungal activity [150].

## 5. Conclusions

The collagen-based fertilizers have proved that they are a good candidate to replace conventional chemical fertilizers. They offer the advantage of recovering a valuable by-product (the collagen) from leather waste, and of transforming it into a valuable product.

When the collagen source is chromium-tanned leather, chromium content should be carefully checked, as its concentration in the soil must be in agreement with the imposed regulations.

The synthesized biopolymers have shown good fertilization, comparable, or even better, than the conventional chemical fertilizers, for a large range of crops (tomato, beans, peas, soybean, wheat, ornamental plants, etc.).

The nutrient release tests have demonstrated that, by adjusting the collagen content in the fertilizer, and by cross-linking the collagen with synthetic polymers, the N release can be controlled and slowed.

Another advantage given by the use of a controlled release fertilizer is that the loss of nutrients caused by leaching in the inferior layers of the soil or ground water is reduced, avoiding water contamination with nitrate.

The soil humidity is maintained for a longer time, because of the collagen capacity to retain large amounts of water, and in this way, the irrigation frequency is reduced.

The cross-linking of natural polymers with polyelectrolytes (polyacrylamide) has ameliorated the soil quality, by preventing crust formation, a fact which helps the plant seeding, especially for plants with small seeds.

## Figures and Tables

**Table 1:** Comparison between different biopolymer-based fertilizers obtained from leather waste.

Fertilizer Type	Soil Type	Crop	Rate	Comments
Poultry manure [82]	Central Italy—unspecified type	tomato	100 kg N/ha	Does not fulfill the crop demand in nutrients
Poultry manure and by-product from leather factory [82]	Central Italy—unspecified type	tomato	100 kg N/ha	The fertilizer gave the same efficacy as the mineral fertilizer.
Organic fertilizer by-product from leather factory [82]	Central Italy—unspecified type	tomato	100 kg N/ha	The fertilizer gave the same efficacy as the mineral fertilizer.
Mineral fertilization [82]	Central Italy—unspecified type	tomato	100 Kg N/ha and 200 Kg N/ha	The fertilizer gave the same efficacy as the fertilizers by-products from leather factory
Collagen-based biofertilizer [83]	stagnic albeluvisol, Romania; degraded soil classified as dusty clay soil	soybean	10 kg fertilizer/m^2^ 20 kg fertilizer/m^2^	The second rate provided only a slightly higher production (about 0.2%), compared with the first-rate, and both gave about 20% more productivity, compared to unfertilized soil.
Collagen-based biofertilizer; collagen extracted from wet white leather waste [84]	Neutral or slightly alkaline soil	peas	0.25–0.50 kg fertilizer/m^2^	Good results on soil quality improvement and crop quantity.
Collagen extracted from wet blue leather [85]	Yellow-Red Latosol, clayey texture, Oxisol, pH = 5.9	bean plants cultivated after the growth of elephant grass on the soil fertilized with collagen	4, 8, 16, or 32 t collagen/ha	Results similar to mineral fertilization.

**Table 2:** Composition of different biopolymer-based fertilizers obtained from leather waste.

Fertilizer Type	% N	P, (Expressed as % P_2_O_5_)	K, (Expressed as % K_2_O)	Other Components	Comments	Reference
NPK, universal fertilizer	26	13	6	0.004% Cu, 0.037% Fe, 0.03% Mn, 0.0015% Mo, 0.015% Zn	it is used for any type of culture	Produced by Azomures S.A. [86]
Radicon N30	30	−	−	−	7.5% NO_3_ − N + 7.5% NH_4_-N + 15% urea	[82]
Urea	46	−	−	−	−	[40]
Ammonium sulfate	21	−	−	−	−	[40]
Ammonium nitrate	30.5	−	−	−	−	[40]
Floranid	32	−	−		Low solubility material containing (3% urea-N; 29% IBDU—isobutilidenediurea -N)	[40]
Fertilizer by-product from leather factory	5	−	−	C/N = 5.4	The fertilizer with higher N content gave better results for tomato crop.	[82]
Organic fertilizer by-product from leather factory	8	−		C/N = 2.8		[82]
Gelatine based fertilizer; gelatine extracted from leather waste	43.84 (weight)	−	Not specified	7.72% C; 40.26% O; 1.76% Na; 0.35% Al; 0.2% Si; 0.05% S; 5.28% Cl; 0.54% Ca		[87]
Collagen-based biofertilizer; collagen extracted from leather waste	11.14	2.43	3.77	0.127% Mg	pH of aqueous extract 7–7.5	[83]
Collagen based fertilizer cross-linked with different polymers: (a) collagen hydrolysate with nutrients encapsulated as reference sample (b) collagen hydrolysate functionalized with P(SSNa-co-GMAx) copolymer (c) collagen hydrolysate functionalized with poly-acrylamide (d) collagen hydrolysate functionalized with functionalized with starch	10.55 10.14 12.13 8.29	7.67 6.75 5.79 5.54	10.62 8.21 8.40 10.07	(expressed as % TOC) 45.2 37.56 48.1 64.32	pH = 7.2 pH = 6.87 pH = 6.76 pH = 6.20	[77]
Collagen extracted from wet blue leather	14.6	2.6	0.014		Collagen was applied on a soil having the pH 5.9, and only minor changes in the soil pH were observed, in the range of 5.9–6.1	[85]

## Data Availability

Not applicable.
